# Jingfang granules protects against intracerebral hemorrhage by inhibiting neuroinflammation and protecting blood-brain barrier damage

**DOI:** 10.18632/aging.205854

**Published:** 2024-05-28

**Authors:** Yanling Li, Qingying Yu, Huiyuan Peng, Xie Mingjun, WenHua Xu, Tingting Zheng, Tingting Zhao, Mengyao Xia, Jibiao Wu, Pantelis Stavrinou, Roland Goldbrunner, Yicheng Xie, Guimin Zhang, Yu Feng, Yongxia Guan, Feng Zheng, Peng Sun

**Affiliations:** 1 School of Pharmacy, Shandong University of Traditional Chinese Medicine, Ji’nan, China; 2 Guangdong Provincial Key Laboratory of Translational Cancer Research of Chinese Medicines, Joint International Research Laboratory of Translational Cancer Research of Chinese Medicines, International Institute for Translational Chinese Medicine, School of Pharmaceutical Sciences, Guangzhou University of Chinese Medicine, Guangzhou, China; 3 Department of Pharmacy, Zhongshan Hospital of Traditional Chinese Medicine, Zhong Shan, China; 4 College of Chinese Medicine, Shandong University of Traditional Chinese Medicine, Ji’nan, China; 5 Prevention and Treatment Center, Shenzhen Hospital of Integrated Traditional Chinese and Western Medicine, Shenzhen, China; 6 Shandong University of Traditional Chinese Medicine, Ji’nan, China; 7 Innovation Research Institute of Chinese Medicine, Shandong University of Traditional Chinese Medicine, Ji’nan, China; 8 Department of Neurosurgery, Center for Neurosurgery, Faculty of Medicine and University Hospital, University of Cologne, Cologne, Germany; 9 The Children’s Hospital, Zhejiang University School of Medicine, National Clinical Research Center for Child Health, Hangzhou, China; 10 State Key Laboratory of Generic Manufacture Technology of Chinese Traditional Medicine, Lunan Pharmaceutical Group Co., Ltd., Linyi, China; 11 Department of Neurosurgery, The Second Affiliated Hospital of Fujian Medical University, Quanzhou, China

**Keywords:** jingfang granules, intracerebral hemorrhage, metabolomics, network pharmacology, neuroinflammation

## Abstract

Intracerebral hemorrhage (ICH) can induce intensive oxidative stress, neuroinflammation, and brain cell apoptosis. However, conventional methods for ICH treatment have many disadvantages. There is an urgent need for alternative, effective therapies with minimal side effects. Pharmacodynamics experiment, molecular docking, network pharmacology, and metabolomics were adopted to investigate the treatment and its mechanism of Jingfang Granules (JFG) in ICH. In this study, we investigated the therapeutic effects of JFG on ICH using behavioral, brain water content and Magnetic resonance imaging experiments. However, the key active component and targets of JFG remain unknown. Here we verified that JFG was beneficial to improve brain injury after ICH. A network pharmacology analysis revealed that the anti-inflammatory effect of JFG is predominantly mediated by its activation of the phosphatidylinositol 3-kinase (PI3K)/AKT pathway through Luteolin, (+)-Anomalin and Phaseol and their targeting of AKT1, tumor necrosis factorα (TNF-α), and interleukin-1β (IL-1β). Molecular docking analyses revealed an average affinity of −8.633 kcal/mol, indicating a binding strength of less than −5 kcal/mol. Metabolomic analysis showed that JFG exerted its therapeutic effect on ICH by regulating metabolic pathways, such as the metabolism of taurine and hypotaurine, biosynthesis of valine, leucine, and isoleucine. In conclusion, we demonstrated that JFG attenuated neuroinflammation and BBB injury subsequent to ICH by activating the PI3K/Akt signaling pathway.

## INTRODUCTION

Stroke is divided into ischemic stroke (IS) and intracerebral hemorrhagic stroke (ICH). ICH is a cerebrovascular disease that seriously threatens human health. Due to the high incidence, disability and mortality rates, stroke is regarded as the primary cause of death in my country [1, 2]. ICH accounts for 10–15% of all strokes, and may cause brain damage, including primary and secondary damage [3]. The primary injury is defined as the damage to adjacent brain tissue caused by mechanical compression and the mass effect of the hematoma in the early stage of ICH, and the secondary injury is defined as the subsequent nerve damage due to the components of the hematoma and its metabolites [4]. At present, the clinical treatment for ICH is primarily based on the removal of thrombus, intraventricular hemorrhage, intracranial pressure, and the control of systemic hypertension. However, effective acute or preventive therapeutic methods are not available [5]. Numerous studies have shown that the causes of ICH are complex, such as amyloid angiopathy, tumors, and vascular malformations [6]. Additionally, the pathophysiology of ICH is also complex, in which neuroinflammation represents a critical defense of the host to the secondary brain injury that follows ICH. Elevated thrombin levels, hemolysis, and increased pro-inflammatory cytokines may aggravate tissue damage after ICH, while increased anti-inflammatory cytokines may have protective effects on the brain in ICH [7, 8].

The multicomponent and multitarget characteristics of traditional Chinese medicine and its compounds enable it to better act in different stages of ICH. Eleven traditional Chinese medicines are included in Jingfang Granules (JFG), i.e., *Schizonepeta tenuifolia* (Benth.) Briq., S*aposhnikovia divaricate* (Turcz.) Schischk., *Notopterygium incisum* K.C. Ting ex H.T.Chang, *Heracleum hemsleyanum* Diels, *Ligusticum striatum* DC., *Bupleurum chinense* DC., *Platycodon grandifloras* (Jacq.) A.DC., Citrus *aurantium* L., *Peucedanum praeruptorum* Dunn, *Poria cocos* (Schw.) Wolf., and *Glycyrrhiza uralensis* Fisch. ex DC [9]. JFG have the potential to induce perspiration, release exterior, disperse wind and dispel dampness, which can significantly reduce the duration of illness of patients with cold and can significantly improve their cold symptoms [10]. According to the “Property and Flavor-Herb-Meridian” network diagram constructed by Chen [11], it can be seen that the medicinal materials in JFG are mainly hot, bitter, and sweet, most of which are belonged to lung, spleen, and liver channels. This aligns with traditional Chinese medicine approaches to stroke treatment, which is mostly sweet and hot, and the meridians are liver, spleen, heart, lung and kidney [12]. Meanwhile JFG contain *Ligusticum chuanxiong* Hort, *Glycyrrhiza uralensis* Fisch and other traditional Chinese medicine commonly used for treating stroke [13].

Modern research shows that JFG can reduce the activity of interleukin-6 (IL-6), IL-1β, TNF-α, and other inflammatory factors, exert its antipyretic and antiviral activities [14–17] and has obvious therapeutic effect on liver fibrosis induced by CCL4 through anti-oxidation and regulating TGFB/SMAD4 signaling pathway [18]. At the same time, it has been proved that it can activate PI3K-Akt signaling pathway to contribute to the management of alcoholic liver disease [19], and can alleviate the acute renal injury caused by aristolochic acids by regulating the AKT MDm2 p53 pathway and inhibiting apoptosis [20]. At the same time, it was also found that JFG could significantly increase the activities of superoxide dismutase (SOD), catalase (CAT) and the content of glutathione (GSH), and at the same time reduce the levels of ROS and malondialdehyde (MDA), play an anti-oxidative stress role, thus playing a role in myocardial protection [21]. Among 11 traditional Chinese medicines, obvious therapeutic effects of Chuanxiong, Bupleurum, Licorice, and Poria on stroke have been observed [22]. The active ingredients senkyunolide A, Z-ligustilide [16], Ligusticum, Ligustrazine, Ligustylid [23], ferulic acid, and Senkyunolide H [24] in Chuanxiong can dramatically inhibit the production of pro-inflammatory mediators in mouse BV-2 microglia after lipopolysaccharide (LPS) stimulation, obviously impair the aggregation of thrombosis and platelet, as well as blood viscosity to improve cerebral microcirculation, significantly reduce infarct volume, neurological dysfunction, BBB disruption, and cerebral edema [25–28]. The active ingredient Saikosaponin A in Bupleurum can significantly improve post-stroke depression (PSD)-like behavior, inhibit neuronal apoptosis, increased the levels of brain-derived neurotrophic factor and Bcl-2 in the hippocampus of PSD rats, and reduced the levels of Bax and caspase-3 [29]. Guizhi Fuling Pill has been found to decrease the levels of nitric oxide (NO), inducible NO synthase (iNOS), and cyclooxygenase-2 (COX-2) expression in LPS-induced BV-2 microglia. It also reduces the ratio of Bax/Bcl-2 and the expression of caspase-3 protein, thus showing good anti-apoptosis effect [30]. Liquiritigenin (LQ), the active ingredient in licorice, can activate the Keap1 /Nrf2 antioxidant pathway, inhibit endoplasmic reticulum stress, and maintain the integrity of the BBB [31]. However, whether JFG can be used to treat ICH has not been studied.

In this study, the application of network pharmacology and metabolomics was implemented to investigate the possibility that JFG may provide a defensive response 118 to secondary damage resulting from ICH, including potential mechanisms related to 119 inflammation and integrity of the BBB. Moreover, we aimed to determine whether JFG 7 120 has the potential to be an effective remedy for ICH.

## MATERIALS AND METHODS

### Reagents and animals

JFG (0012007009) was bought from Shandong New Times Pharmaceutical Co., Ltd. Primary antibodies of anti-β-actin (GB12001, Servicebio, Wuhan, China), anti-GAPDH (GB12002, Servicebio, Wuhan, China), anti-MMP9 (GB11132, Servicebio, Wuhan, China), anti-PI3K (GB11525, Servicebio, Wuhan, China), anti-AKT (GB13427, Servicebio, Wuhan, China), anti-p-AKT (AF0016, Affinity Biosciences, USA), anti-IL-1β (AF5103, Affinity Biosciences, USA), anti- Occludin (DF7504, Affinity Biosciences, USA), anti- Claudin-5 (AF5216, Affinity Biosciences, USA), and anti-TNF-α (GB11188, Servicebio, Wuhan, China). Evans Blue (CAS: 314-13-6) was purchased from Bioengineering (Shanghai) Co., Ltd. (Shanghai, China). The Collagenase IV (C8160) and rabbit fluorescein conjugated antibody were purchased from Servicebio (Wuhan, China). Adult male rats (Sprague-Dawley rat, 300 ± 20 g) were purchased from Pengyue Laboratory Animal Breeding Co., Ltd. (Jinan, Shandong, China).

### ICH induction and drug administration

The rats were housed under conditions of constant temperature, humidity, and a 12/12-h light/dark cycle, with food and water available, and deprived of food and water for six hours before surgery. Rats were anaesthetized using isoflurane and secured to a rat stereotaxic apparatus for experimentation. A burr hole with a diameter of 1 mm was created at the given coordinates in relation to the bregma: 0.2 mm anterior, 3.5 mm left lateral, 6 mm deep [32]. Subsequently, a 1 μl micro syringe (Gaoge, Shanghai, China) was employed to introduce 1μl saline with 0.2 U collagenase IV (C8160, Solarbio, Beijing, China) at a speed of 0.1 μl/min for a duration of 10 minutes. The needle was left in place for 10 minutes, before the micro syringe was removed, then the micro syringe was pulled. Following the surgery, the aperture was sealed with bone wax and the scalp was closed utilizing sutures. A rat thermostatic blanket was implemented to maintain the body temperature at 37° C throughout the procedure. The rats in the sham group operated with the similar procedures without collagenase infusion. After the surgery, a behavioral scoring was performed according to the Longa five-point scale, and a score of 1-3 indicated that the modeling was successful. Male Sprague Dawley (SD) rats were administered JFG and saline by intragastric injection and randomly divided into 4 experimental groups: Sham + saline (n = 6), ICH + saline (n = 6), ICH + JFG (9.36 g/kg, n = 6), and ICH + JFG (14.04g/kg, n = 6) groups.

### Beam balance test (BBT)

BBT was measured as described previously [33]. Take a 2.0 × 1.5 × 80 cm wooden strip, one end is fixed, the fixed end is set with a baffle to prevent the rat from escaping, the other end is suspended at a height of 50 cm above the ground, and the wooden strip is horizontal. A 40 × 80 cm foam board was placed on the ground under the wooden strip to prevent the rat from falling from the wooden strip. Place the rat in the center of the wooden bar and start timing. Observe the posture of the rat on the wooden bar, the holding time, and the left and right deviations when falling. The experiment was terminated when the rat fell from the wooden bar, and the experiment was also terminated when the longest was 2 min and was scored. Balance beam test scoring standards are as follows: 1 point: stand firmly on the wooden bar, no shaking, for 2 minutes; 2 points: stand firmly on the wooden bar, shake left and right, but not slide down, lasting 2 minutes; 3 points: Stand on the wooden bar, slide to one side, but do not fall for 2 minutes; 4 points: stand on the wooden bar for more than 30 seconds and fall off the wooden bar within 2 minutes; 5 points: try to stand firmly on the wooden bar, But fall off within 30 s; 6 points: No standing ability and falling off from the wooden bar. from the wooden bar immediately.

### Elevated body swing test (EBST)

The EBST has been modified several times since it was first reported. We adopted the improved method in this experiment [34]. The tail of the rat was held with one hand and lifted so that the head of the rat is about 10 cm from the ground. At this point, the rat swayed and twisted to one side in an attempt to escape. When the angle between the longitudinal axis of the rat’s head and the vertical axis was greater than or equal to 90°, the suspension torsion was recorded once and the torsion deflection (left/right) was recorded at the same time. Place the rat on the ground and let it rest for 2 min before performing the next suspension twist. The experiment ended when a total of 20 suspension twists were recorded. Normal animals will go up to the left and right same times and will not deviate to one side. Therefore, the bias should be zero. On the contrary, animals with unilateral hemorrhagic brain injury often showed marked deviation to one side.

### Magnetic resonance imaging (MRI) and measurement

MRI was measured as described previously [35]. The animal’s body temperature was kept at 37° C, and to monitor the respiratory rate, the sensor was placed under the rat chest wall. The isoflurane inhalation was performed to anesthetize the animals during the surgery and examinations (inhalation gas: 20% O_2_/80% air mixture, isoflurane concentration of 2.5% during the anesthesia, and 0.8% to 1% during MRI detection). The rats were randomly selected based on the MRI detection on the 3rd day using a Bruker Biospec 94/20 USR small animal magnetic resonance imaging system (Bruker BioSpin MRI, Ettlingen, Germany). The volume of the posterior ventricle was observed to evaluate the actual degree of brain damage. The parameters for Image Acquisition are as follows: TR: 3140 ms, TE: 37 ms, FOV: 40×40 mm, inversion angle: 180°, bandwidth: 130 Hz/px, matrix: 320×240, layer thickness: 1.0 mm. After image processing, IKT-SNAP 3.2.0 and Python 3.7 were used to analyze the area and volume of the regions affected by bleeding in MR T2.

### Measurement of brain edema

On day 3 after ICH in rats, the rats were executed and their brains were immediately divided into hemorrhagic hemisphere, contralateral hemisphere and cerebellum. Each tissue was then weighed individually to obtain the wet weight (WW). The tissues were then dried at 100° C for 24 hours to obtain the dry weight (DW), and the brain water content was then assessed by the formula: (WW-DW)/(WW) × 100%.

### Blood-brain barrier permeability

BBB permeability was determined by measuring the extravasation of Evans blue (EB) as described previously [36]. A 2% EB solution in sterile saline (4 ml/kg body weight) was injected intraperitoneally (i.p.) into the rats. After 24 hours, the brain tissue was stored at 1100 μL PBS. Then centrifuge the sample at 6000rpm for 30 minutes at 4° C. Collect the supernatant and mix it with an equal volume of trichloroacetic acid ethanol (1:3). Incubate the sample overnight at 4° C and centrifuge for 30 minutes (6000rpm, 4° C). The content of EB dye was measured by spectrophotometry at 620nm and quantified from the standard curve.

### Western blot assay

The rats were anesthetized on the third day after ICH, and the left hemisphere of the brain was stored at -80° C until it was used. Firstly, prepare a complete lysis solution using RIPA (G2002, Servicebio, China) and phosphorylated protease inhibitor (BL615, Biosharp, China) of which 1 μL phosphorylated protease inhibitors A and B are added to each 100 μl RIPA. And remove the stored cerebral hemorrhage tissue from the -80° C refrigerator, melt it at 4° C, wash it 2-3 times with pre cooled PBS, wash the blood stains, and weigh it. Next, add a complete cracking solution with a volume of 10 times the sample, and use the uniform slurry machine at 6500 rpm for 30s-40s. Finally, place it on ice and let it stand for 30 minutes, vortex every 5 minutes to fully crack it. Centrifuge at 14000 rpm for 10 minutes, retain the supernatant, and store at -80° C. The protein solution was mixed with loading buffer reagent and then denatured by boiling at 100° C for 5 minutes. Then 50 mg of protein were loaded on an SDS-PAGE gel for electrophoresis, the condition was set to a voltage of 70V for 30 minutes, and then uses 110V voltage to bring the protein strip to the bottom of the separation glue. Then transferred to a PVDF membrane (Pall Corporation, NY, USA, G6015-0.45), the condition was set to a current of 400 mA for 90 min at an ice-water bath. Subsequently the membrane was blocked 3% bovine serum albumin V and incubated overnight at 4° C with the primary antibodies listed below: anti-β-actin (1:1000;), anti-GAPDH (1:2000), anti-MMP9 (1:1000), anti-PI3K (1:1000), anti-AKT (1:1000), anti-p-AKT (1:1000), anti-IL-1β (1:1000), anti- Occludin (1:1000), anti- Claudin-5 (1:1000), and anti-TNF-α (1:1000). Suitable secondary antibodies (1:3000, Biosharp, China) were chosen and left on the membrane for 30 minutes at room temperature. The blot bands were then viewed using an ECL reagent (Biosharp, China, BL520A). Non-saturated bands were selected and quantified using Image J software (Image J 1.4, NIH, USA).

### Measurement of NO level

The rats were anesthetized on day 3 after ICH. NO levels in serum were determined spectrophotometrically. The level of NO was assessed through the Griess reaction (Nanjing Jiancheng, China) as described previously [37]. Griess Reagent I and Griess Reagent II were added in equal volume, and then the absorbance at 540 nm was detected by enzymic labeler. Finally, NO content was calculated by nitrite standard curve.

### Immunofluorescence staining

Coronal brain sections were blocked with 5% bovine serum albumin at room temperature for 2 h. Each section was 10 μm thick. Then incubated with Iba (1, 1:1000, Servicebio) antibody at 4° C overnight. The sections were washed with PBS, incubated with secondary antibody, and then autofluorescence quencher was added before staining the nuclei with DAPI and covering the sections with anti-fade fluorescence mounting solution (G1401; Servicebio, Wuhan, China). Finally, the sections were examined by fluorescent microscopy (Nikon Eclipse C1, NIKON, Japan). The number of positive cells was quantified with the ImageJ software.

### Statistical analysis

All experimental data were analyzed and plotted using GraphPad Prism 8 software, and the results were expressed as mean ± standard error of the mean (Mean ± S.E.M.). Means of two groups were compared using Student’s t-test and one-way ANOVA was used for comparing multiple groups. Statistical significance was set at P < 0.05.

### Network pharmacology analysis of JFG

In the traditional Chinese medicine systems pharmacology database (TCMSP) database (https://www.tcmsp-e.com/#/home), using criteria of oral bioavailability (OB) ≥ 30% and drug-like property (DL) ≥ 0.18, the effective components of 11 traditional Chinese medicines (including Notopterygii Rhizoma et Radix, Aurantii Fructus, Poria cocos, Saposhnikovia divaricata, Platycodon grandiflorus, Heracleum hemsleyanum Diels, Peucedanum praeruptorum Dunn, Glycyrrhiza uralensis Fisch, Radix Bupleuri, Ligusticum chuanxiong Hort and Nepeta cataria L) were searched, and obtain corresponding potential target information. Limit “Homo sapiens” and “Reviewed” in UniProt database (https://www.uniprot.org/) was used to provide up-to-date genetic information on targets. The Cytoscape software was conducted to construct a medicinal material-ingredient-target network. We use ‘hemorrhagic stroke and intracerebral hemorrhage‘ as the keyword in GeneCards(https://www.genecards.org/), Therapeutic Target Database (TTD) (http://db.idrblab.net/ttd/), and Online mendelian inheritance in man (OMIM) (https://www.omim.org/) drug databases for ICH-related targets. The targets that intersect with JFG were identified using Venn2.1. The protein-protein interaction network was constructed using the String database, and the core targets were obtained by topological analysis. The Metascape database (https://metascape.org/gp/index.html) was used for Gene Ontology (GO) enrichment and Kyoto Encyclopedia of Genes and Genomes (KEGG) pathway enrichment analyses to predict the mechanism of action.

### Molecular docking of potential active ingredients to core target proteins

The PubChem database was used to obtain the structures of the individual compounds, then the 3D molecular structures of the receptor were downloaded by entering the number into the RCSB PDB database(https://www.rcsb.org/, AKT1 ID: 4gv1, Resolution: 1.49 Å; TNF ID: 1vyr, Resolution: 0.9 Å; TP53 ID: 2j21, Resolution: 1.6 Å; IL6 ID: 1alu, Resolution: 1.9 Å; VEGFA ID:1mkk, Resolution: 1.32 Å; CASP3 ID: 4ps0, Resolution: 1.63 Å; IL1B ID: 5r85, Resolution: 1.44 Å; JUN ID: 2g01, Resolution: 3.5 Å; ESR1 ID: 5n10, Resolution: 1.60 Å; MAPK3 ID: 4qtb, Resolution: 1.4 Å). In this study, we used CBDock 2 (https://cadd.labshare.cn/cb-dock2/php/index.php)for docking.

### Metabolomics analysis

The 25mg sample was extracted with 500 μL extraction solution (methanol: acetonitrile: water = 2:2:1). Then, homogenize for 4 minutes, ultrasound in the ice water bath for 5 minutes, and repeat the above steps 3 times. The samples were then incubated at -40° C for 1 h and centrifuged at 12000 rpm for 15 min at 4° C. The supernatant of all samples is mixed in equal amounts to prepare samples for quality control (QC). The LC-MS/MS analysis was performed using Waters ACQUITY UPLC BEH Amide (2.1 mm × 100 mm, 1.7 μm) coupled to a Q Exactive HFX mass spectrometer (Orbitrap MS, Thermo). The mobile phase was 25 mmol/L ammonium acetate and 25 ammonium hydroxide, aqueous solution (pH = 9.75) (A) and acetonitrile (B), the gradient elution procedure is: 0-0.5 min, 95% B; 0.5-7 minutes, 95% -65% B; 7-8 minutes, 65% B-40% B; 8-9 minutes, 40% B; 9-9.1 minutes, 40% -95% B; 9.1-12 minutes, 95% B, the temperature of the injector was 4° C, and the injection volume was 2 μL. Using Thermo Q Active HFX mass spectrometer, sheath gas flow: 30 Arb, auxiliary gas flow: 25 Arb, capillary temperature: 350° C, full MS resolution: 120000, MS/MS resolution: 7500, collision energy under NCE mode: 10/30/60, spray voltage: 3.6 kV (positive) or -3.2 kV (negative). Then import the data into SIMCA 14.1. Principal component analysis (PCA) and orthogonal partial least-squares discriminant analysis (OPLS-DA) were performed after data standardization. Differential metabolites with variable importance in projection (VIP) greater than 1 and Student’s t-test (P-value) lower than 0.05 were used for metabolic pathway analysis.

## RESULTS

### JFG could alleviate ICH-induced neurological deficits

**Figure 1:**
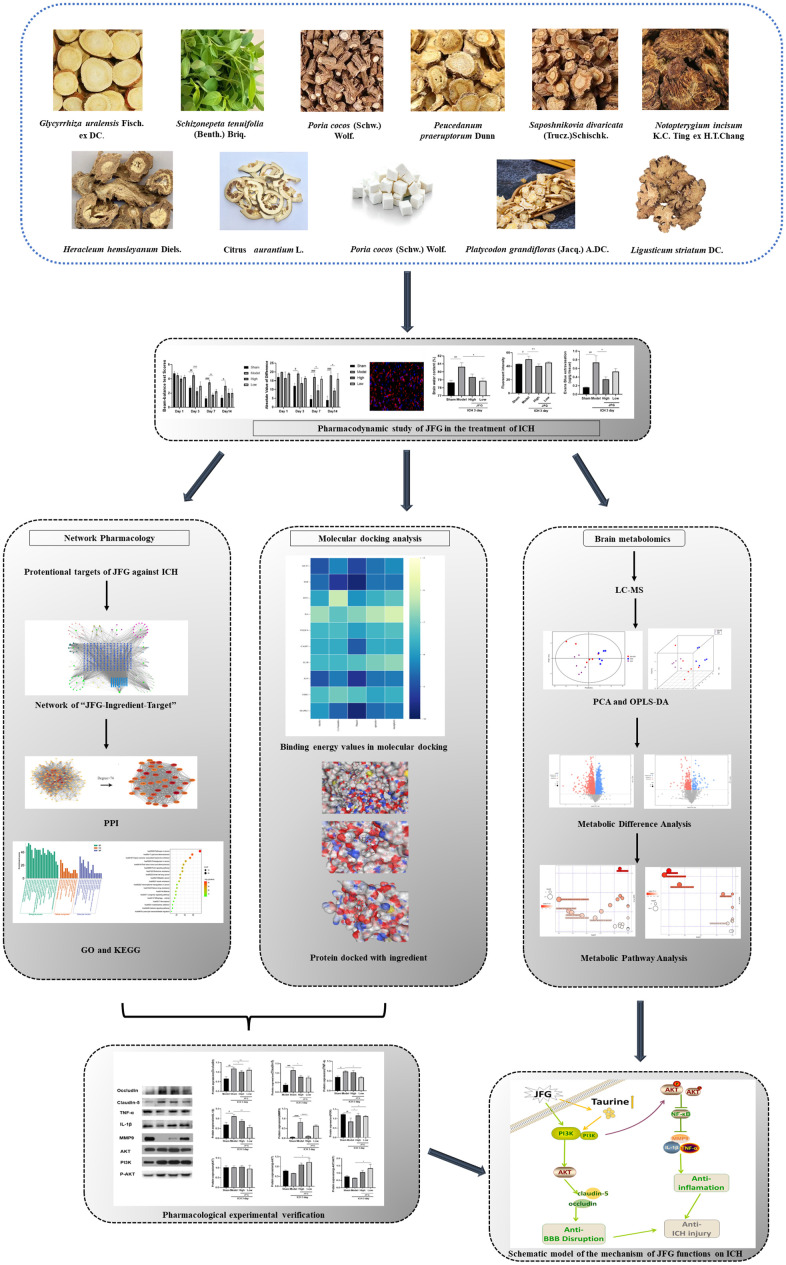
Schematic model of the mechanism of JFG functions on ICH.

**Figure 2:**
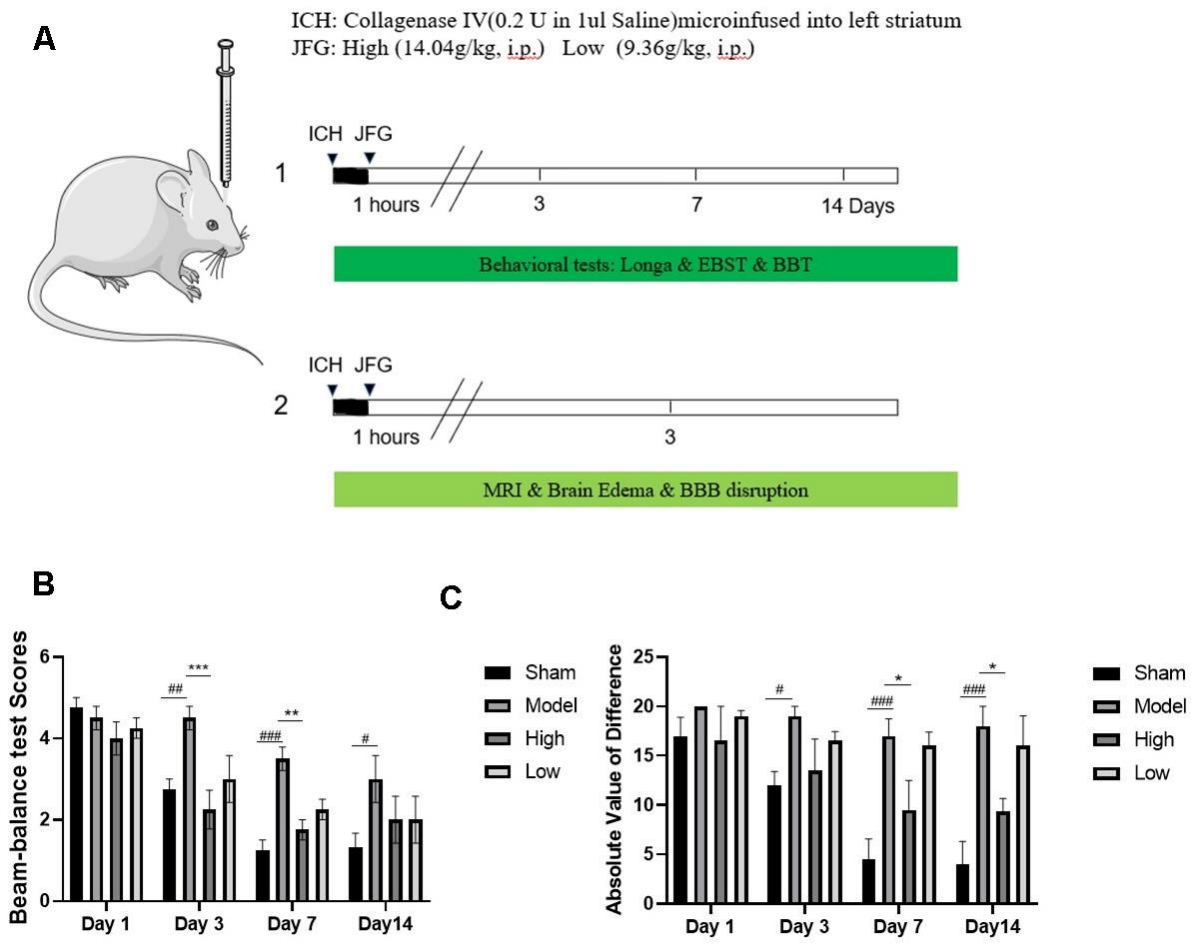
**JFG attenuated neurological deficits.** At 1, 3, 7, and 14 days post ICH induction, JFG treatment resulted in significant reduction of neurological deficits. (**A**) Schematic model of the mechanism of JFG functions on ICH. (**B**) Representative bar chart illustrating outcomes of the BBT analysis. (**C**) Representative bar graph showing the results of EBST assay. Values are presented as means ± S.D. ***P < 0.001; **P < 0.01; *P < 0.05, compared with Model group; ^###^P < 0.001; ^##^P < 0.01; ^#^P < 0.05, compared with the Sham group. (n = 6, 1,3,7,14 days).

**Figure 3:**
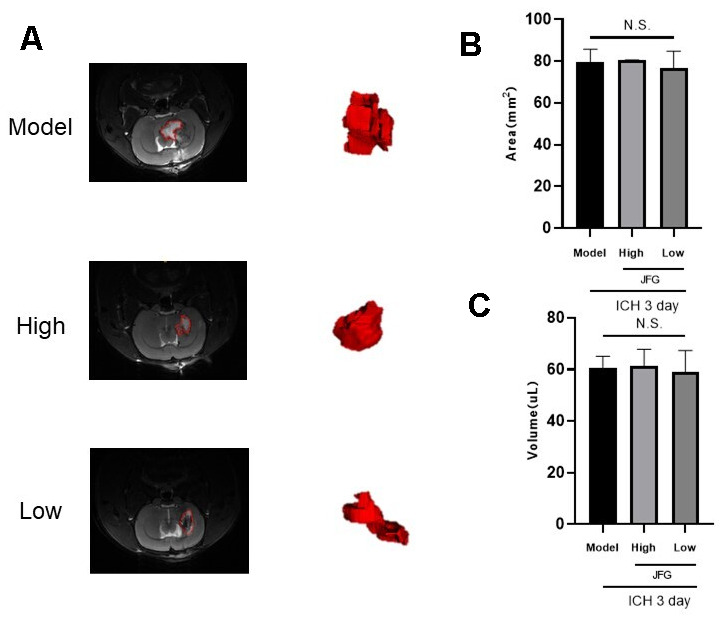
(**A**, **B**) T2-weighted MRI imaging of rats with ICH. (**A**) T2 weighted imaging was used to observe intracerebral hemorrhage areas (The red dotted line indicates the size of the hematoma). (**B**) intracerebral hemorrhage volume. (**C**) Representative bar graphs showing the area of hematoma (left panel) and the volume (right panel). Values are presented as means ± S.D. ***P < 0.001; **P < 0.01; *P < 0.05, compared with Model group; ^###^P < 0.001; ^##^P < 0.01; ^#^P < 0.05, compared with the Sham group. (n = 6, 3 days).

We assessed the defensive properties of JFG regarding cerebral damage in rats afflicted with ICH. Figure 1 illustrates the diagram of the experimental design in this study. Figure 2A shows the schematic model of the mechanism of JFG functions on ICH. In our experiment, the scores of BBT increased markedly at 3, 7 and 14 days (P < 0.01, P < 0.001, P < 0.05) (Figure 2C) after ICH induction. The Absolute Value of the difference increased markedly at 3, 7 and 14 days (P < 0.05, P < 0.001, P < 0. 001) (Figure 1D and Table 1) after ICH. The delivery of JFG could significantly reduce the BBT scores (P < 0.001, day 3; P < 0.01, day 7) and the absolute value of the difference at 14.04g/kg (P < 0.05, day 7 and 14) (Figure 2B, 2C and Table 2). Collagenase injection vastly induced hematoma 3 days after ICH induction, however, neither dose of JFG had a significant impact on the area or volume of the hematoma. (Figure 3A–3C).

**Table 1:** Behavioral score of EBST.

**Groups**	**n**	**Day1**	**Day3**	**Day7**	**Day14**
Sham	6	17.00±3.83	12.00±2.83	4.50±4.12	4.00±4.00
Model	6	20.00±0.00	19.00±2.00^#^	17.00±3.47^###^	18.00±3.47^###^
High	6	16.50±7.00	13.50±6.40	9.50±5.98^*^	9.33±2.31^*^
Low	6	19.00±1.16	16.50±1.92	16.00±2.83	16.00±5.30

Values are presented as means ± S.D. *P < 0.05, compared with Model group; ^###^P < 0.001 ^#^P < 0.05, compared with the Sham group. (n = 6, 1,3,7,14 days).

**Table 2:** Behavioral score of BBT.

**Groups**	**n**	**Day1**	**Day3**	**Day7**	**Day14**
Sham	6	4.75±0.50	2.75±0.50	1.25±0.50	1.33±0.58
Model	6	4.50±0.58	4.50±0.58^##^	3.50±0.58^###^	4.33±1.155^#^
High	6	4.00±0.82	2.25±0.82^***^	1.75±0.50^**^	2.00±1.00
Low	6	4.25±0.50	3.00±1.16	2.25±0.50	2.00±1.00

Values are presented as means ± S.D. ***P < 0.001; **P < 0.01; *P < 0.05, compared with Model group; ^###^P < 0.001; ^##^P < 0.01; ^#^P < 0.05, compared with the Sham group. (n = 6, 1,3,7,14 days).

### JFG could alleviate ICH-induced BBB destruction and brain edema

**Figure 4:**
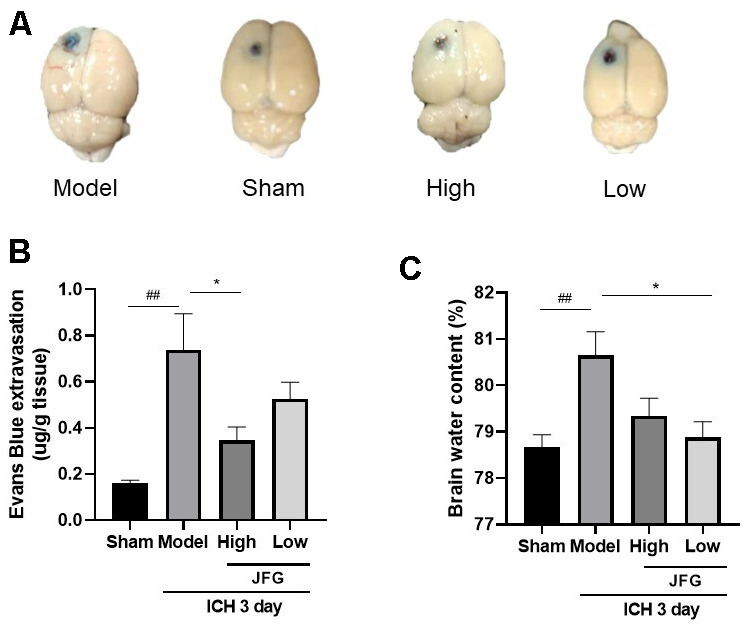
**JFG may alleviate damage to the BBB and swelling of the brain caused by ICH.** (**A**) Represents the surface view of EB extravasation at 3days after ICH in each group. (**B**) Representative bar chart demonstrating the condensed findings of the extravasation of EB dye on day 3 post-ICH. (**C**) Representative bar graph showing the statistical results of the brain water content using the wet/dry weigh method on 3 day after ICH. Values are presented as means ± S.D. ***P < 0.001; **P < 0.01; *P < 0.05, compared with Model group; ^###^P < 0.001; ^##^P < 0.01; ^#^P < 0.05, compared with the Sham group. (n=6,1,3,7,14days).

The extravasation of EB dyes demonstrated a clear increase following ICH (P < 0.01, 3 days), while JFG administration (14.04 g/kg) could significantly reduce the EB dyes leakage resulting from ICH (P < 0.05, 3 days) (Figure 4A, 4B). After ICH, BWC significantly increased (P < 0.01, 3 days). JFG at 9.36 g/kg could significantly reduce the increased BWC induced by ICH (P < 0.05, 3 days) (Figure 4C and Table 3).

**Table 3:** Brain water content ($\bar{X}\pm \text{SEM}$).

**Groups**	**Brain water content**
Sham	78.67 ± 0.2667
Model	80.64 ± 0.5167^##^
JFG-High	79.34 ± 0.3827
JFG-Low	78.880 ± 0.3361^*^

### JFG decreased the migration of activated microglia into the perihematomal tissue after ICH

**Figure 5:**
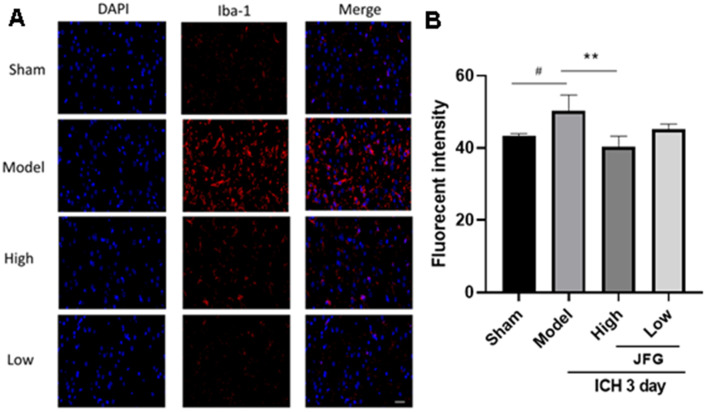
**JFG decreased the migration of activated microglia into the perihematomal tissue after ICH.** (**A**) Representative images showing the staining of Iba-1 tissues from indicated group of rats. Scale bar: 100 μm (**B**) Bar graph represents the summarized results of the fluorescent intensity. Values are presented as means ± S.D. ***P < 0.001; **P < 0.01; *P < 0.05, compared with the Model group; ^###^P < 0.001; ^##^P < 0.01; ^#^P < 0.05, compared with the Sham group. (n = 6, 3 days).

The degree of microglia activation is proportional to Iba-1 expression (Figure 5A).

The staining of rat brain sections showed that a large number of Iba-1 positive cells could be seen around the hematoma of ICH group and the model group (P < 0.05, 3 days) (Figure 5B), indicating the recruitment of activated microglia. However, after JFG treatment (14.04g/kg), the level of Iba-1 positive cells significantly decreased compared to those of rats in both of the ICH and the model groups (P < 0.01, 3 days) (Figure 5B). The results showed that JFG treatment significantly reduced the activation of microglial cells in perihematomal tissue after ICH.

### Prediction of the possible herb-compound-target-pathway interaction associated with the inhibitory effect of JFG on stroke using Network pharmacology

**Figure 6:**
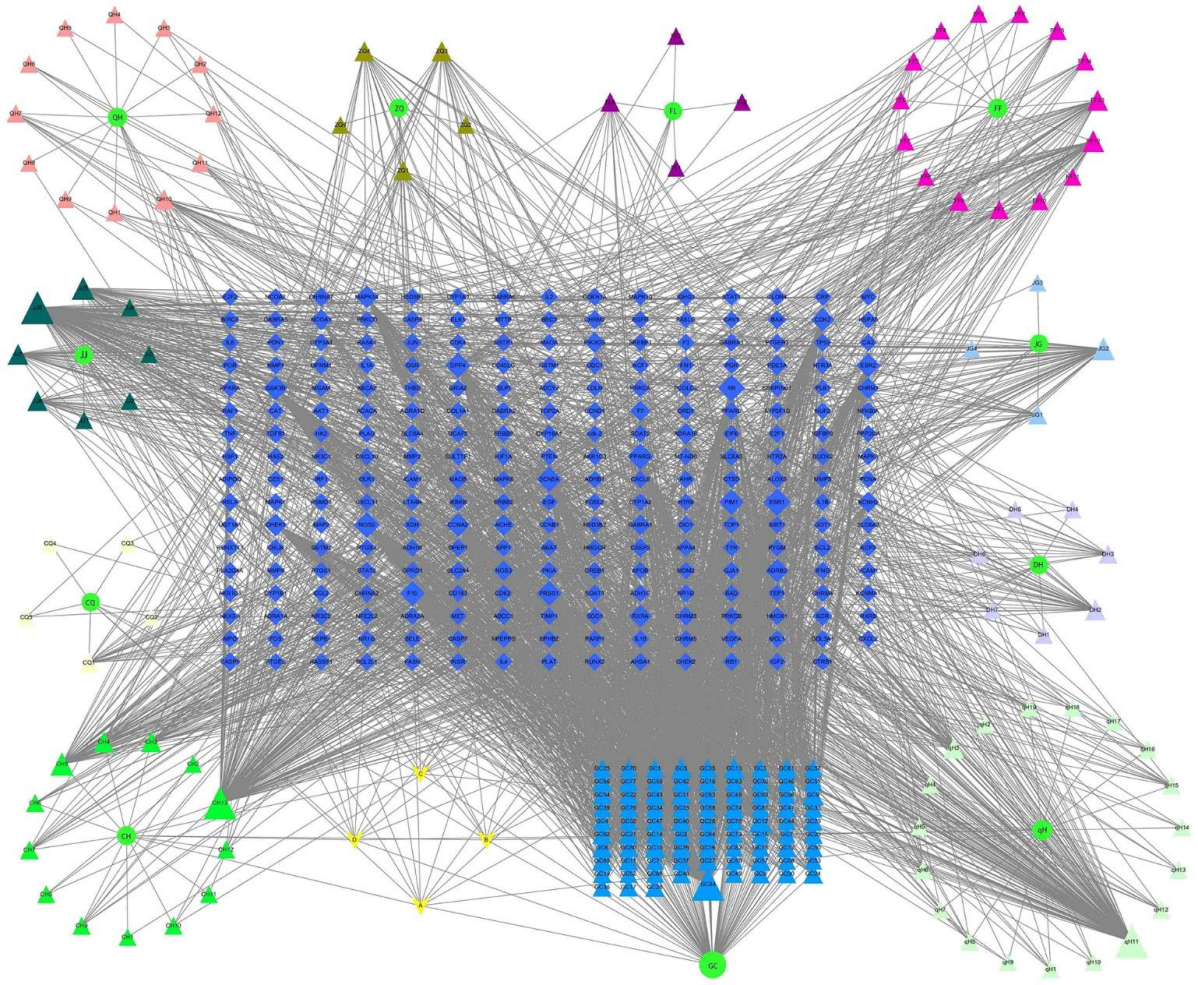
**Network of “JFG-Ingredient-Target”.** Circle represent 11 herbs in JFG, triangles represent active components, diamonds represent target genes, A, B, C, D represent the common ingredients of 11 medicinal materials in JFG. *Notopterygium incisum* Ting ex H. T. Chang (Qianghuo, QH), *Aurantii Fructus* (Zhiqiao, ZQ), *Poria cocos* (Fuling, FL), *Saposhnikovia divaricata* (Turcz.) Schischk. (Umbelliferae) Schischk. (Fangfeng, FF), *Platycodon grandiflorus* (Jacq.) A.DC. (Jiegeng, JG), *Heracleum hemsleyanum Diels* (Duhuo, DH), *Peucedanum praeruptorum* Dunn (Qianhu, qH), *Glycyrrhiza uralensis* Fisch. (Gancao, GC), *Radix Bupleuri* (Chaihu, CH), *Ligusticum chuanxiong* Hort. (Chuanxiong, CQ), *Nepeta cataria L.* (Jingjie, JJ).

**Figure 7:**
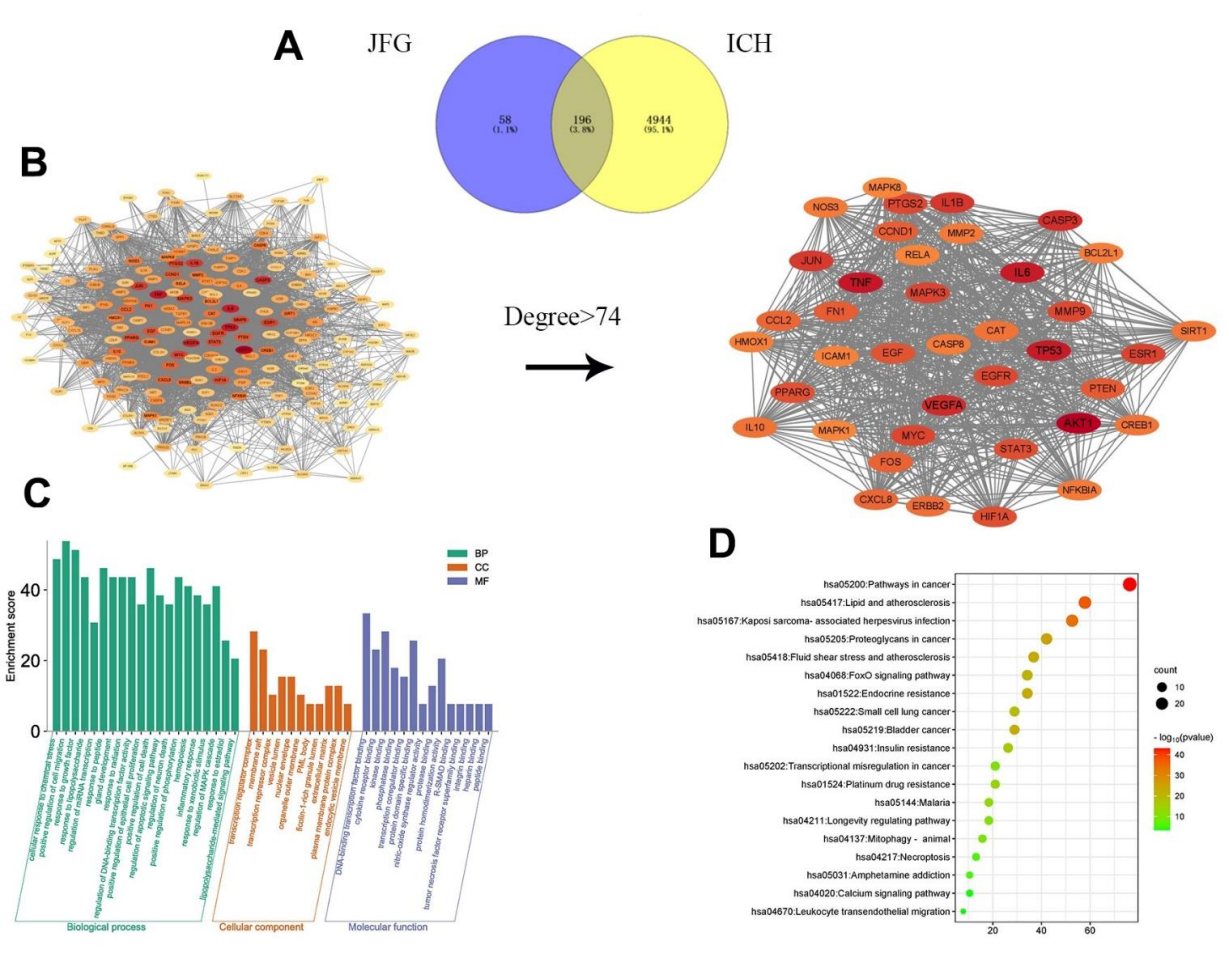
**Network pharmacology prediction of JFG treatment for stroke.** (**A**) The intersection of JFG targets and disease targets. (**B**) PPI analysis of JFG and stroke intersection targets. (**C**) GO enrichment analysis. (**D**) KEGG enrichment analysis.

To investigate the diverse constituents, multiple targets, and numerous pathways of JFG in combating ICH, network pharmacology was conducted. First, the active ingredients of JFG were extensively searched in the TCMSP database, 159 active ingredients of 11 traditional Chinese JFG medicines and 253 corresponding target information were obtained. Then the Cytoscape 3.8.2 software was applied to construct the ‘JFG-Ingredients-Target’ Network (Figure 6). Meanwhile, 5140 genes which were dysregulated in ICH were extracted from the OMIM, TTD and GeneGards databases, and 196 intersection targets of JFG and ICH were discovered by Venn 2.1.0 (Figure 7A). The protein-protein interaction (PPI) network was constructed using the String database. According to the network topology analysis of Degree > 74, 39 key target points of 11 Chinese herbs acting on ICH were obtained (Figure 7B). The core targets were subjected to GO and KEGG pathway enrichment analyses, GO analysis can describe the biological function of genes, which is composed of molecular function (MF), biological process (BP), and cell component (CC). The results showed that MF was mainly composed of DNA-binding transcription factor binding, cytokine receptor binding, kinase binding, etc. Among BP, the top ones are cellular response to chemical stress, positive regulation of cell migration, response to growth factor, etc. In CC analysis, transcription regulator complex, membrane raft, vesicle lumen, etc. The top channels in KEGG are pathway in cancer, Lipid and atherosclerosis, proteoglycans in cancer, fluid shear stress and atherosclerosis (Figure 7C, 7D). In conclusion, the network pharmacology analysis indicated that the therapeutic effect of JFG on ICH may involve in the PI3K/AKT signaling pathway and the inflammatory mediators, such as MMP9, TNF-α, and IL-1β.

### Molecular docking experiments

**Figure 8:**
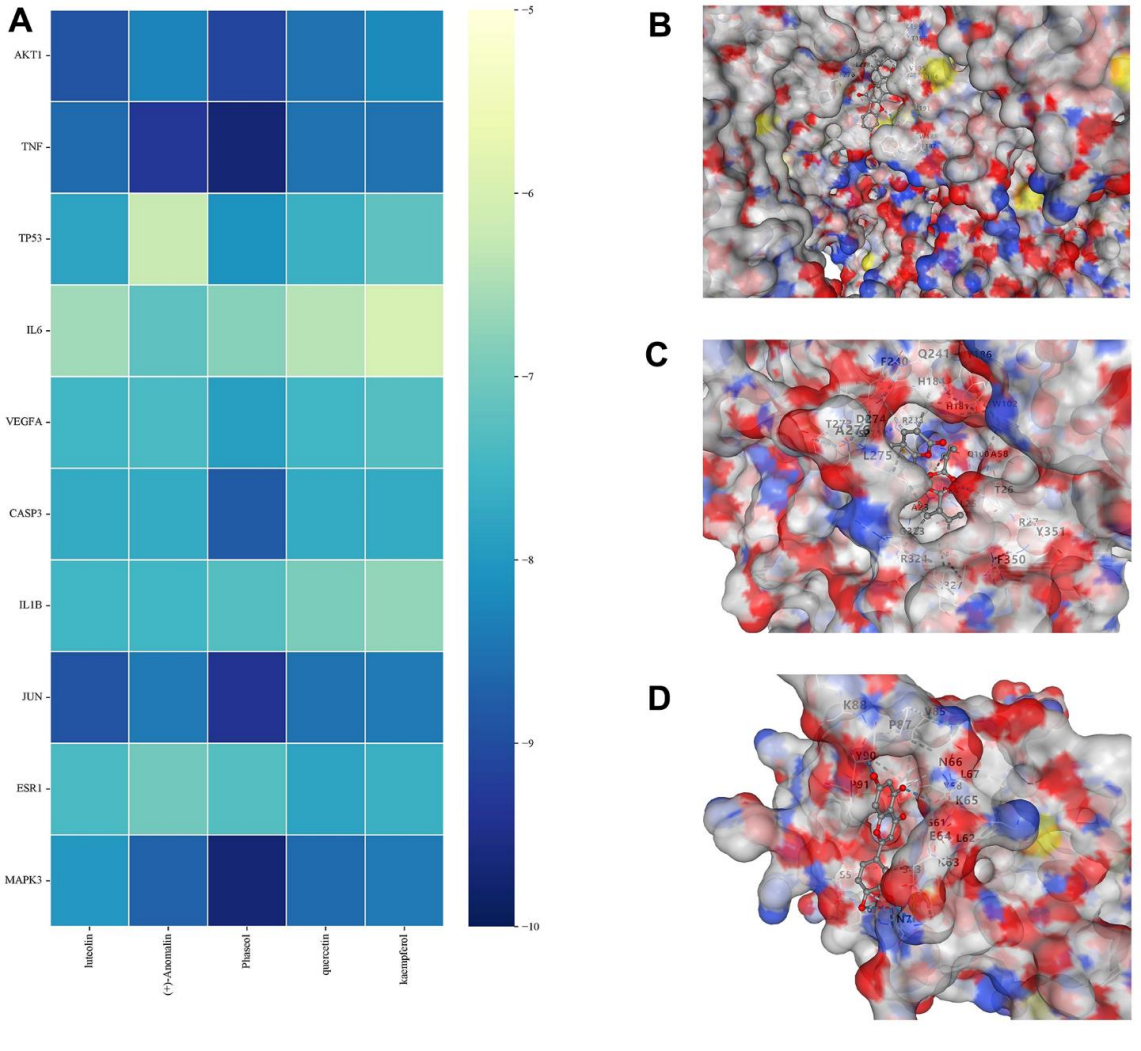
**Molecular docking results.** (**A**) Binding energy values in molecular docking. (**B**) AKT1 docked with Phaseol. (**C**) TNF-α docked with (+)-Anomalin. (**D**) IL-1B docked with luteolin.

First, five core components (luteolin, (+)-Anomalin, Phaseol, quercetin and kaempferol) obtained during the construction of network pharmacology were selected. The lower molecular docking binding energy, the smaller molecules can bind to proteins. Results showed that Luteolin, (+)-Anomalin and Phaseol were strongly associated with AKT1, IL-1β and TNF-α (Figure 8). Therefore, these substances are considered to be effective substances and main targets of JFG in the treatment of neuroinflammation.

### JFG could trigger PI3K / AKT pathway activation

**Figure 9:**
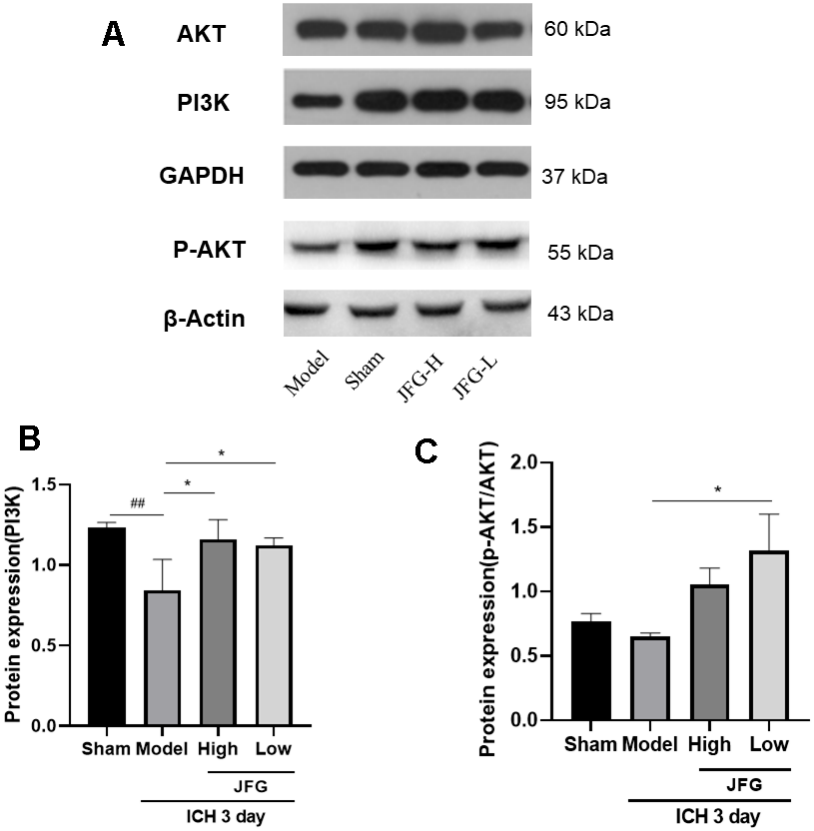
**Verification of the protein expression in the tissues from differentially treated rats.** (**A**) Representative blots showing the expression of PI3K, p-AKT, and AKT in the tissues from indicated groups of rats. (**B**, **C**) The bar graphs represent the summarized results of the protein expression of PI3K (**B**) and the ratio of p-Akt/Akt. Values are presented a means ± S.D. ***P < 0.001; **P < 0.01; *P < 0.05, compared with the Model group; ^###^P < 0.001; ^##^P < 0.01; ^#^P < 0.05, compared with the Sham group. (n = 6, 3 days).

As showed in Figure 9A, both doses of JFG could markedly upregulate the expression of PI3K expression (P < 0.05) (Figure 9B). And JFG (9.36 g/kg) could dramatically increase the ratio of p-AKT/AKT (P < 0.05) (Figure 9C).

### JFG could down-regulate the expression of pro-inflammatory cytokines

**Figure 10:**
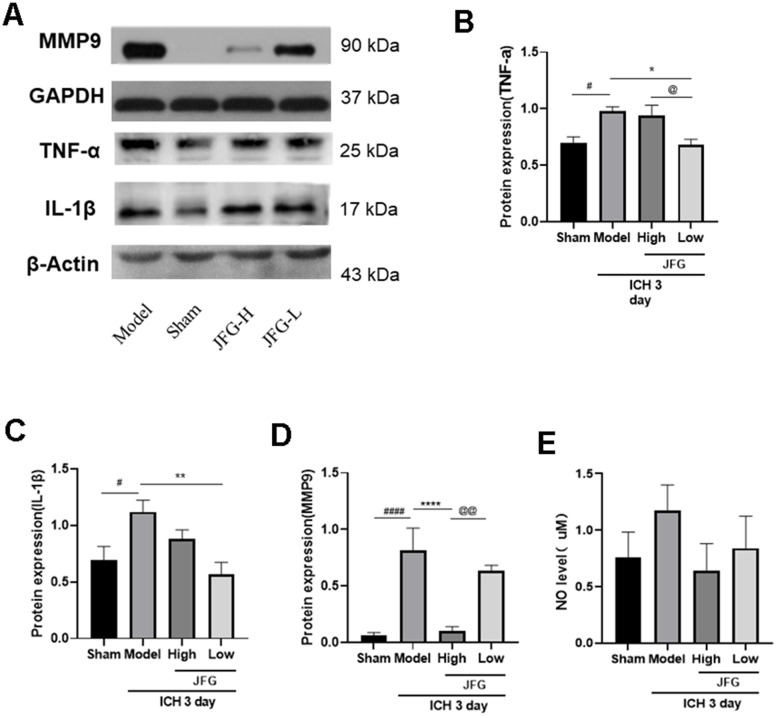
**Verification of the protein expression in the tissues from differentially treated rats.** (**A**) Representative blots showing the expression of TNF-α, IL-1β, and MMP9 in the tissues from indicated groups of rats. (**B**–**D**) The bar graphs represent the summarized results of the protein expression of TNF-α (**B**), IL-1β (**C**), MMP9 (**D**), and the NO content in serum (**E**). Values are presented a means ± S.D. ***P < 0.001; **P < 0.01; *P < 0.05, compared with the Model group; ^###^P < 0.001; ^##^P < 0.01; ^#^P < 0.05, compared with the Sham group; ^@@^P < 0.01; ^@^P < 0.05, compared with the JFG-H group. (n = 6, 3 days).

We performed Western blot to examine the impact of JFG on the production of inflammatory cytokine production 3 days after ICH. The results indicated that after ICH, the production of inflammatory cytokines (TNF-α, IL-1β, MMP9) increased markedly (Figure 10A), and JFG (14.04 g/kg) could significantly reduce MMP9 production (P < 0.001) (Figure 10D), and JFG (14.04 g/kg) could reduce NO levels, but there was no statistical difference. (Figure 10E). Similarly, we also observed the significantly decreased production of TNF-α (P < 0.05) (Figure 10B) and IL-1β (P < 0.01) (Figure 10C) in the ICH rats after JFG treatment at 9.36 g/kg.

### JFG could up-regulate expression of Claudin-5 and Occludin

**Figure 11:**
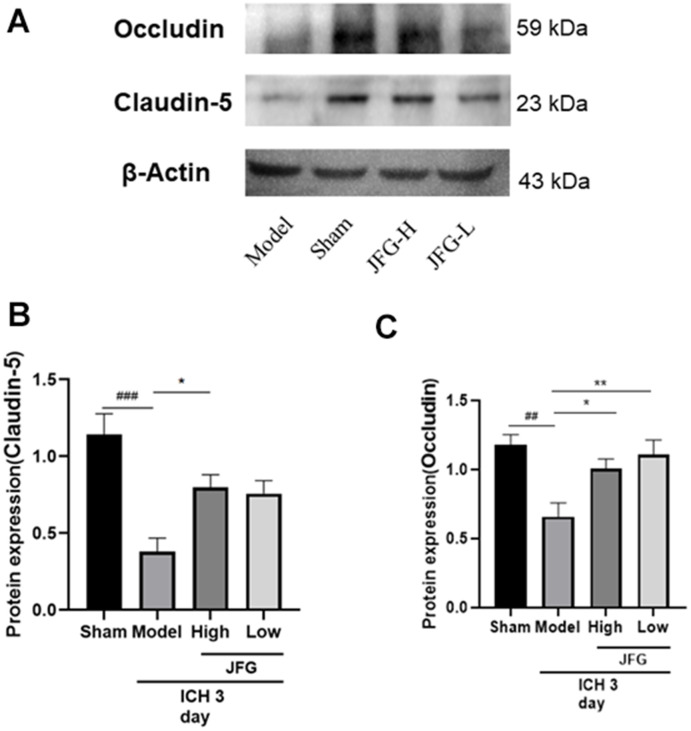
**Verification of the protein expression in the tissues from differentially treated rats.** (**A**) Representative blots showing the expression of Claudin-5 and Occludin in the tissues from indicated groups of rats. (**B**, **C**) The bar graphs represent the summarized results of the protein expression of Claudin-5 (**B**) and Occluding (**C**). Values are presented a means ± S.D. ***P < 0.001; **P < 0.01; *P < 0.05, compared with the Model group; ^###^P < 0.001; ^##^P < 0.01; ^#^P < 0.05, compared with the Sham group. (n = 6, 3 days).

We performed Western blot to examine the impact of JFG on the production of Claudin-5 and Occludin production 3 days after ICH. The results showed that after ICH (Figure 11), the significantly increased production of Claudin-5(P < 0.05, 3 days) (Figure 11B) in the ICH rats after JFG treatment at14.04 g/kg. Similarly, we also observed JFG (9.36 g/kg) could significantly increase Occludin production (P < 0.01) (Figure 11C).

### Metabolomics analysis revealed variations in metabolites in ICH tissues among the JFG, model, and control groups

**Figure 12:**
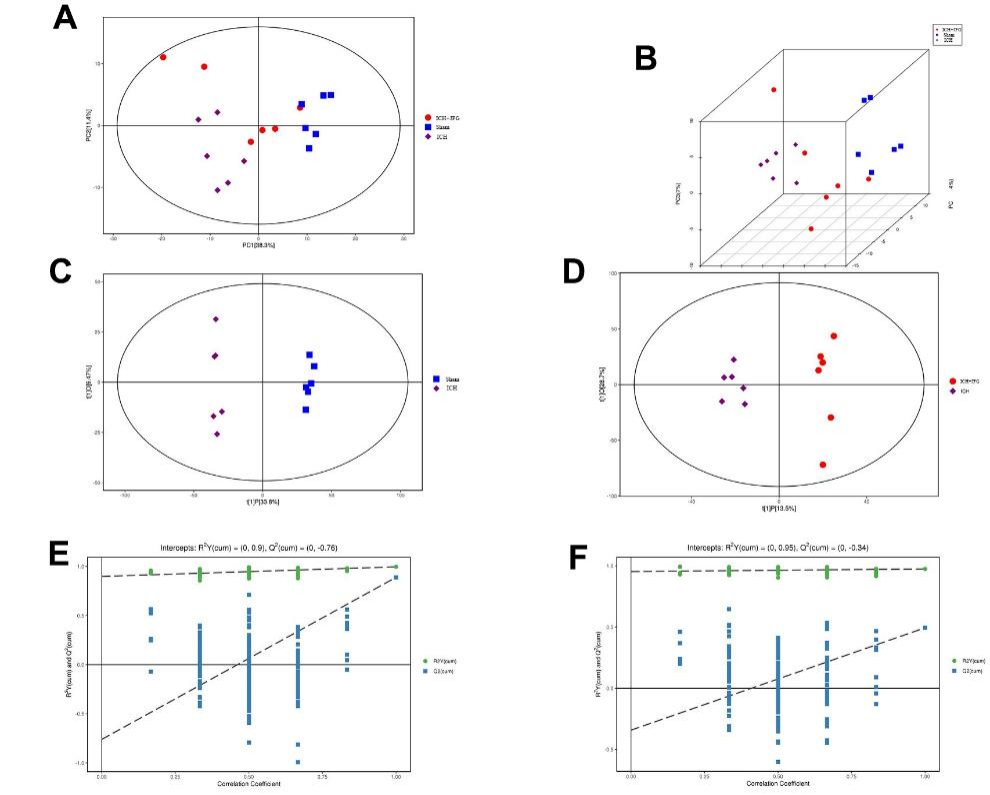
**Multivariate statistical analysis of metabolites.** (**A**) Represents the Score scatter plot of the PCA model; (**B**) Represents 3D Score scatter plot of the PCA model. (**C**, **D**) Represent the score scatter plots of the OPLS-DA model for the Comparisons between the Sham group and ICH group or the ICH + JFG group and the Model group (**D**). (**E**, **F**) Represent permutation plot tests of the OPLS-DA model for the comparisons between the Sham group and the Model group or the ICH + JFG group and the Model group (**F**).

**Figure 13:**
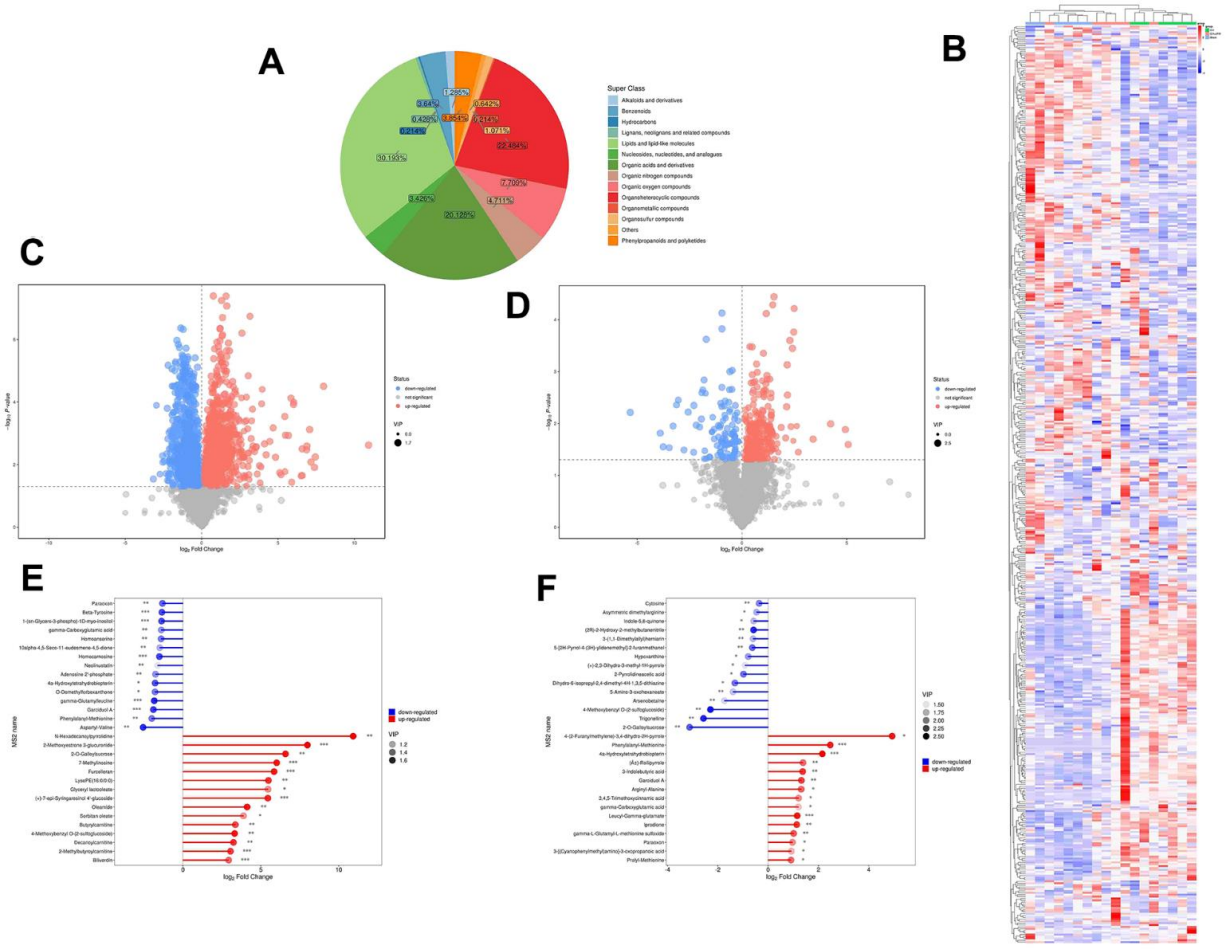
**Metabolite comprehensive analysis.** (**A**) Representative Pie chart showing the metabolite classification and proportion. (**B**) The Heatmap showing the results of the hierarchical clustering analysis for all groups. (**C**, **D**) Significantly up-regulated metabolites are represented in red, while those significantly down-regulated are represented in blue, unchanged metabolites are shown in gray. The comparisons between ICH and control groups (**C**) or ICH + JFG and ICH groups (**D**) are showed. (**E**, **F**) The first 15 most up- or down-regulated metabolites are displayed. The comparisons between ICH and control groups (**E**) or ICH + JFG and ICH groups (**F**) are showed.

**Figure 14:**
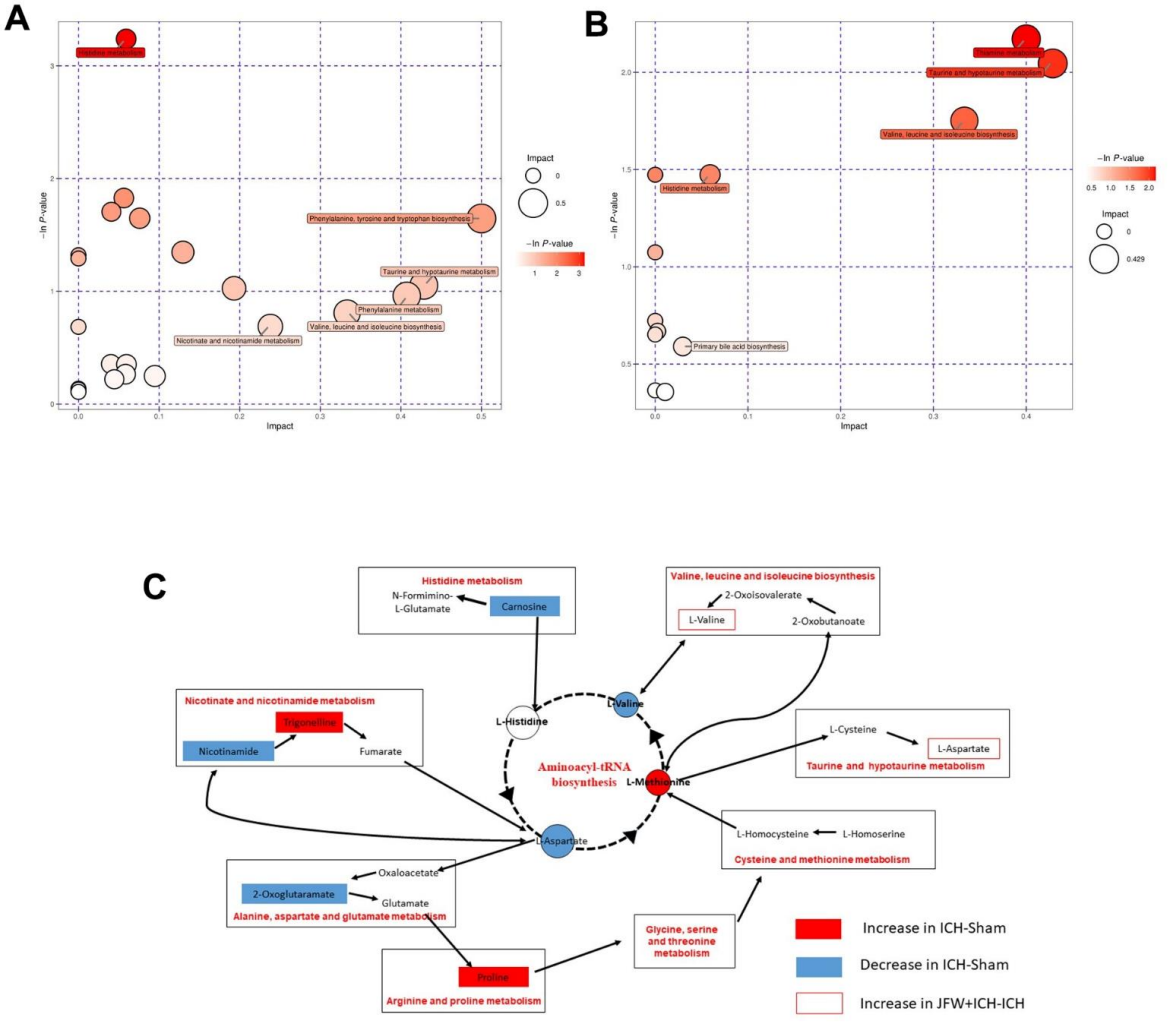
**Comprehensive analysis of metabolic pathways.** Each bubble in the bubble map represents a metabolic pathway. (**A**, **B**) The comparisons between ICH and Sham groups (**A**) or ICH + JFG and ICH groups (**B**) are showed. (**C**) Represents the metabolic pathways found by metabolomics analysis.

To comprehend the metabolic impacts of JFG on ICH, distinct metabolites in ICH tissue samples from rats were identified and analyzed. In basic data analysis, PCA was employed to analyze data related to quality control, and the stability and reproducibility of our system were showed in the score graphs (Figure 12A, 12B). We employed OPLS-DA for multivariate analysis to assess the metabolic profiling data of the Sham, ICH and ICH+JFG group. A replacement test with the number of tests set to 200 was performed, and it was found that the R^2^ and Q^2^ slopes of the ICH group vs Sham group as well as the JFG group vs ICH group were all greater than 0, and all of them had a Q^2^ regression line with the intersection point with the vertical axis less than 0, which indicated that the model was not overfitted, and the data were reliable. Meanwhile, after OPLS-DA analysis, the JFG and ICH groups were able to be significantly separated, indicating that the endogenous metabolites of the two groups differed significantly, and that JFG administration may have altered some endogenous metabolites in ICH rats (Figure 12C–12F). For metabolomic analysis, there are 240 metabolites in the Sham, ICH and ICH + JFG groups, the most abundant metabolites identified were lipids and lipid-like molecules, followed by organoheterocyclic compounds, organic acids and derivatives, etc. (Figure 13A). Compared to the model group, a significant reversal trend of many metabolites, including Paraoxon, gamma-carboxyglutamic acid, Garciduol A, Phenylalanyl-Methionine, 4a-Hydroxytetrahydrobiopterin, Deoxycytidine, L-Valine, Taurine, etc., was found in the JFG treatment group (Figure 13B–13F and Table 4). To further elucidate the fundamental mechanism behind the variation in metabolic products, we conducted an analysis of KEGG (https://www.kegg.jp/)) and MetaboAnalyst (https://www.metaboanalyst.ca). Many markedly changed pathways, such as taurine and hypotaurine metabolism, valine, leucine, and isoleucine biosynthesis, primary bile acid biosynthesis, and Purine metabolism, etc. were discovered between the model and JFG groups at the metabolomic level (Figure 14A, 14B). These findings facilitated the identification of a metabolomics signature of JFG biologic actions. Following our analysis of the metabolic pathway, we established the metabolic disorder diagram associated with the ICH model. (Figure 14C).

**Table 4:** Information on differential metabolites reversed by JFG.

**NO**	**Differential metabolites**	**ICH vs. Sham**				**JFG vs. ICH**			
		**VIP**	**P-Value**	**Fold change**	**trend**	**VIP**	**P-Value**	**Fold change**	**trend**
1	5-Aminopentanoic acid	1.42	0.000882	1.28	up	1.89	0.0220	0.88	down
2	Deoxycytidine	1.31	0.003636	1.34	up	1.77	0.0198	0.78	down
3	L-Valine	1.63	0.000004	0.74	down	1.71	0.0413	1.08	up
4	Cytosine	1.43	0.000832	1.42	up	1.96	0.0082	0.78	down
5	N-[(Ethoxycarbonyl)methyl)-p-menthane-3-carboxamide	1.39	0.002914	0.65	down	2.09	0.0125	1.30	up
6	4-Methoxybenzyl O-(2-sulfoglucoside)	1.65	0.003249	9.96	up	2.45	0.0045	0.20	down
7	2-Pyrrolidineacetic acid	1.12	0.016344	2.40	up	2.04	0.0405	0.51	down
8	(+)-2,3-Dihydro-3-methyl-1H-pyrrole	1.32	0.022177	2.44	up	1.50	0.0454	0.53	down
9	5-[2H-Pyrrol-4-(3H)-ylidenemethyl]-2-furanmethanol	1.49	0.000200	1.76	up	2.06	0.0039	0.65	down
10	Indole-5,6-quinone	1.46	0.000581	2.07	up	1.70	0.0242	0.67	down
11	5-Amino-3-oxohexanoate	1.43	0.000686	4.09	up	1.66	0.0076	0.38	down
12	beta-Zearalenol	1.12	0.018936	0.69	down	1.51	0.0340	1.48	up
13	N-Acetylserine	1.24	0.016139	0.63	down	1.93	0.0163	1.36	up
14	(2R)-2-Hydroxy-2-methylbutanenitrile	1.61	0.000002	3.12	up	2.36	0.0010	0.67	down
15	Allysine	1.33	0.018538	0.51	down	1.85	0.0141	1.35	up
16	9-Hydroxybenzo[a]pyrene-4,5-oxide	1.42	0.001995	0.52	down	1.94	0.0129	1.71	up
17	Beta-Guanidinopropionic acid	1.43	0.011651	0.54	down	1.45	0.0329	1.37	up
18	Trigonelline	1.50	0.005828	6.62	up	2.36	0.0060	0.17	down
19	Asymmetric dimethylarginine	1.56	0.000018	2.18	up	1.75	0.0245	0.73	down
20	2-O-Galloylsucrose	1.64	0.003142	95.62	up	2.11	0.0044	0.11	down
21	Taurine	1.00	0.032002	0.83	down	1.55	0.0421	1.15	up
22	Formiminoglutamic acid	1.58	0.000003	0.41	down	1.54	0.0454	1.51	up
23	(Â±)-Rollipyrrole	1.03	0.022125	0.48	down	2.06	0.0026	2.61	up
24	3-(1,1-Dimethylallyl)herniarin	1.37	0.000277	2.10	up	1.72	0.0059	0.66	down
25	PC(20:5(5Z,8Z,11Z,14Z,17Z)/15:0)	1.18	0.023566	0.74	down	1.64	0.0452	1.22	up
26	Linamarin	1.37	0.003692	0.56	down	2.25	0.0016	1.45	up
27	Arsenobetaine	1.14	0.006843	2.30	up	1.58	0.0026	0.30	down
28	2-Keto-glutaramic acid	1.60	0.001385	0.51	down	2.07	0.0220	1.79	up
29	Asitrilobin C	1.62	0.001664	0.47	down	1.86	0.0385	1.40	up
30	p-Cresol glucuronide	1.55	0.003334	0.59	down	1.74	0.0286	1.28	up
31	gamma-Carboxyglutamic acid	1.26	0.004871	0.38	down	1.67	0.0357	2.30	up
32	2-(2-Furanyl)piperidine	1.23	0.012664	0.62	down	1.84	0.0249	1.48	up
33	1-Palmitoylglycerophosphoinositol	1.31	0.006105	0.52	down	1.60	0.0373	1.59	up
34	Iprodione	1.35	0.011306	0.41	down	2.32	0.0017	2.20	up
35	4a-Hydroxytetrahydrobiopterin	1.46	0.010833	0.29	down	2.54	0.0007	4.45	up
36	L-2-Amino-4-methylenepentanedioic acid	1.24	0.011946	0.64	down	2.01	0.0124	1.55	up
37	Phenylalanyl-Methionine	1.34	0.001263	0.25	down	2.39	0.0002	5.52	up
38	Garciduol A	1.52	0.000820	0.27	down	2.27	0.0014	2.50	up
39	Isoniazid alpha-ketoglutaric acid	1.39	0.001324	1.30	up	1.86	0.0089	0.88	down
40	3,4,5-Trimethoxycinnamic acid	1.31	0.001367	0.43	down	1.90	0.0110	2.31	up
41	Na,Na-Dimethylhistamine	1.38	0.001931	0.68	down	1.83	0.0235	1.19	up
42	3-Hydroxychlorpropamide	1.31	0.005652	0.45	down	1.68	0.0378	1.71	up
43	gamma-L-Glutamyl-L-methionine sulfoxide	1.48	0.000023	0.47	down	2.15	0.0024	2.02	up
44	Paraoxon	1.42	0.002421	0.40	down	1.91	0.0201	1.95	up

## DISCUSSION

ICH stands out among stroke, for the severe pathological results it routinely produces. Increasing evidence suggested that apoptosis [38, 39], neuroinflammation [40–43], immunity [44], and the BBB [45] play an important role in ICH. Therefore, although many intervention measures for ICH and its sequelae have been studied (e.g., hemostatic therapy, blood pressure control, hematoma evacuation, and various nerve protection strategies), clinical benefits are still elusive.

In our study, although JFG could not effectively reduce the area of cerebral hemorrhage, it improved cerebral edema in rats with ICH, decreased the expression of the recruitment of activated microglia, effectively reducing BBB damage, and can effectively alleviate the neurological deficit caused by ICH. However, how JFG achieves anti-inflammatory and anti-BBB damage effects through molecular mechanisms *in vivo* remains a key point.

Network pharmacology analysis revealed that JFG exerted its efficacy against ICH via anti-inflammatory and anti-BBB injury by regulating PI3K/AKT pathway. Furthermore, key targets of JFG for treating ICH were identified, including IL-1β, TNF-α, AKT and MMP9. Inflammation and coagulation responses after ICH accelerate the formation of the perihematomal edema in brain, leading to more severe and longer-lasting damage, which is a major contributor to the high mortality of ICH patients. [46]. After brain injury, inflammation M1 microglia, which secrete cytokines IL-1β and TNF-α, are involved in extracellular matrix degradation, cell integrity and BBB damage [47]. Within 7 days of ICH appearance, the phenotype of M1 changes to M2 phenotype, and its secretion of anti-inflammatory cytokines (e.g., IL-4 and IL-10) is critical for the clearance of hematoma, tissue healing, and overall resolution of inflammation [48]. The JFG low dose can significantly reduce the expression of TNF-α and IL-1β after rats ICH, indicating that 9.36 g/kg JFG can inhibit the expression of inflammatory factor and play an ICH protection role. PI3K/AKT is a key regulator of cell behaviors such as apoptosis, proliferation and differentiation [49]. In particular, it plays a neuroprotective role in diseases of the central nervous system, such as Alzheimer’s disease (AD) [50, 51], Parkinson disease (PD) [52, 53], IS [54, 55], and ICH [56–58]. Studies of Bao Yu [59] and Han Cai Ping [60] have shown that PI3K and P-AKT protein expression will show an increase in 6 h after the mouse ICH is made. It began to show a downward trend, only a small amount of expression on the 7th day. Brain edema and neurological deficits caused by ICH may be effectively alleviated by activating the PI3K/Akt signaling pathway, protect the integrity of the BBB, inhibit oxidative stress, and impair ICH-induced brain damage [61]. After JFG was administered, the expression of ICH rats PI3K protein increased significantly, and the p-AKT/AKT ratio increased significantly, indicating that JFG can activate the PI3K/AKT pathway.

BBB mainly maintains the tight connection between adjacent tissue to maintain the cerebral microvascular endothelial cells. The change in the tightly connected to the protein level may cause the BBB permeability to increase the permeability, and thereby exacerbating BBB dysfunction, therefore the integrity of cells is crucial [62]. These important proteins include ZO-1, Occludin, and Claudin-5, which can prevent molecules from flowing from blood flow into the brain and closely related to the increased BBB permeability caused by a variety of diseases [63]. Occludin is the key structural component of BBB, which can regulate the integrity and permeability of BBB, and has become an important focus of BBB damage research. In addition, Occludin is a potential biomarker of bleeding transformation after acute ischemic stroke [64]. Claudin-5 is the richest tight connecting protein BBB. Its dysfunction has confirmed that it is related to neurodegenerative diseases such as Alzheimer’s disease, neuritis diseases such as multiple sclerosis, and mental diseases including depression and schizophrenia [65]. At the same time, improve the expression level of Claudin-5, reduce BBB permeability, improve neural function, and prevent the occurrence of SBI after ICH [66]. Furthermore, MMP9 has been implicated as a pathogenic factor for ICH [67]. A number of studies have shown that the expression of MMP9 significantly increased after the breakdown of the BBB after stroke [68]. Extracellular matrix metalloproteinase induction (EMMPRIN) is a key inflammatory medium in some nervous system diseases, triggering the production of matrix metal protease (MMPS). Studies have shown that Minotin can reduce EMMPRIN, thereby suppressing MMP-9 expression, thereby playing the effect of reducing blood and brain barrier, suppressing neuritis, and playing a protective role in ICH [69]. After 14.04 g/kg’s JFG administration, the expression of ICH rats Occludin and Claudin-5 protein has increased significantly, and the expression of MMP9 is significantly reduced, indicating that the JFG of 14.04 g/kg can play a BBB protection role.

Next, metabolomics analysis was performed to identify differential metabolites between the JFG treatment group and the model group, as well as elucidate the metabolic pathways associated with the protective effect of the JFG on ICH. The results of the metabolomics analysis showed that 222 metabolites are existed in the Sham, ICH, and ICH + JFG groups. Moreover, the metabolic pathway analysis also showed that JFG mainly participates in the metabolic pathways of taurine and hypotaurine through taurine, and exerts its protection effect on the rat from ICH. At the same time, studies have found that taurine is an abundant amino acid in the nervous system may regulate the inflammatory response mediated by microglia through the PI3K/AKT pathway [70], can significantly reduce inflammatory damage in various central nervous system diseases through reducing the expression of TNF-α, IL-1β, and IL-6 [71], and can significantly reduce the brain damage, functional deficit after hemorrhage and reduced brain edema and hemorrhagic lesion volume [72]. Collectively, these results revealed that JFG mitigated ICH-induced brain injury in ICH model rats by inhibiting neuroinflammation and protecting the blood-brain barrier damage.

Our research investigated the new pharmacodynamics of JFG by integrating network pharmacology and metabolomics with pharmacological experiments. It has been demonstrated that JFG can be highly effective in modifying the ICH-induced inflammatory, BBB injury, and neurological deficits, and exert therapeutic effects on ICH through the participation in the taurine and hypotaurine metabolic pathways. These results strongly suggested that JFG could be an effective drug candidate for ICH.

Overall, our results indicate that JFG has potential as a new therapy for ICH. However, there are limitations to the current research. Firstly, our study employed male SD rats as animal models. It is important to note that in clinical epidemiological research, there are also female patients who suffer from ICH. Second, the study solely focused on the impact of JFG in restraining neuroinflammation and safeguarding the BBB against damage three days post ICH, the examination did not include investigation of alterations in markers associated with inflammation over extended time periods. Lastly, although taurine has been shown to potentially modulate microglia-mediated inflammatory responses through the PI3K/AKT pathway reduce the brain damage, functional deficit after hemorrhage, it was not verified whether taurine actually increased in brain samples after JFG administration.

## CONCLUSIONS

In conclusion, our study shows that JFG has the capability of reducing neuroinflammation and BBB injury following ICH by activating the PI3K/Akt signaling pathway. Therefore, JFG could be considered as a potential therapeutic strategy for the management of patients with ICH.
